# MicroRNAs in Serum and Bile of Patients with Primary Sclerosing Cholangitis and/or Cholangiocarcinoma

**DOI:** 10.1371/journal.pone.0139305

**Published:** 2015-10-02

**Authors:** Torsten Voigtländer, Shashi K. Gupta, Sabrina Thum, Jasmin Fendrich, Michael P. Manns, Tim O. Lankisch, Thomas Thum

**Affiliations:** 1 Department of Gastroenterology, Hepatology and Endocrinology, Hannover Medical School, Hannover, Germany; 2 Integrated Research and Treatment Center—Transplantation (IFB-Tx), Hannover Medical School, Hannover, Germany; 3 Institute of Molecular and Translational Therapeutic Strategies, Hannover Medical School, Hannover, Germany; 4 Excellence Cluster REBIRTH, Hannover Medical School, Hannover, Germany; Institute of Hepatology, Foundation for Liver Research, UNITED KINGDOM

## Abstract

**Background and Aim:**

Patients with primary sclerosing cholangitis (PSC) are at high risk for the development of cholangiocarcinoma (CC). Analysis of micro ribonucleic acid (MiRNA) patterns is an evolving research field in biliary pathophysiology with potential value in diagnosis and therapy. Our aim was to evaluate miRNA patterns in serum and bile of patients with PSC and/or CC.

**Methods:**

Serum and bile from consecutive patients with PSC (n = 40 (serum), n = 52 (bile)), CC (n = 31 (serum), n = 19 (bile)) and patients with CC complicating PSC (PSC/CC) (n = 12 (bile)) were analyzed in a cross-sectional study between 2009 and 2012. As additional control serum samples from healthy individuals were analyzed (n = 12). The miRNA levels in serum and bile were determined with global miRNA profiling and subsequent miRNA-specific polymerase chain reaction-mediated validation.

**Results:**

Serum analysis revealed significant differences for miR-1281 (p = 0.001), miR-126 (p = 0.001), miR-26a (p = 0.001), miR-30b (p = 0.001) and miR-122 (p = 0.034) between patients with PSC and patients with CC. All validated miRNAs were significantly lower in healthy individuals. MiR-412 (p = 0.001), miR-640 (p = 0.001), miR-1537 (p = 0.003) and miR-3189 (p = 0.001) were significantly different between patients with PSC and PSC/CC in bile.

**Conclusions:**

Patients with PSC and/or CC have distinct miRNA profiles in serum and bile. Furthermore, miRNA concentrations are different in bile of patients with CC on top of PSC indicating the potential diagnostic value of these miRNAs.

## Introduction

Primary sclerosing cholangitis (PSC) is a rare cholestatic liver disease of unknown aetiology characterised by chronic inflammation and obliterative fibrosis of the intrahepatic and/or extrahepatic bile ducts[1–3]. Patients with PSC are at special risk for hepatobiliary cancer [3]. Up to 14% of patients with PSC develop cholangiocarcinoma (CC) [3–5]. The diagnosis of CC is challenging and often delayed due to the lack of early clinical symptoms and reliable diagnostic markers [1, 3, 6].

Analysis of micro ribonucleic acid (miRNA/miR) patterns has attracted particular interest in hepatology [7–9]. MiRNAs are small, non-coding RNA molecules which regulate gene expression by binding to complementary sites of targeted mRNAs leading to translational repression or degradation [7, 10, 11]. MiRNAs participate in the regulation of multiple cell types under physiological and pathological conditions and are essential in different cellular processes such as development, proliferation, apoptosis, metabolism, morphogenesis, and in diseases [12].

Recently, we showed significant differences of miRNA levels in bile of patients with ischemic type biliary lesion compared to anastomic strictures after liver transplantation[9]. As reliable biomarkers for CC detection in patients with PSC are still missing, we hypothesized that miRNAs in bile or serum may serve as a tumor marker for CC. Therefore, our aim was to evaluate differences in miRNA patterns in serum and bile of patients with PSC, CC complicating PSC (PSC/CC) and CC to further clarify the aetiopathogenesis of these diseases and subsequently to improve the clinical management of these patient cohorts.

## Methods

### Patients

We analyzed serum and bile from consecutive patients with PSC (n = 40 (serum), n = 52 (bile)), patients with CC (n = 31 (serum), n = 19 (bile)) and patients with PSC/CC (n = 12 (bile)) presenting for endoscopic retrograde cholangiography (ERC) to the endoscopic unit of Hannover Medical School in a cross-sectional study between 2009 and 2012. Serum samples from healthy individuals (n = 12) were analyzed as additional control. The screening and study patients had at least a two year follow-up period to exclude patients with undiagnosed tumors. Serum and bile samples were partially obtained from different patients.

The diagnosis of PSC was based on laboratory and clinical findings or typical cholangiographic features (strictures or irregularity of intrahepatic and/or extrahepatic bile ducts) and exclusion of secondary causes for sclerosing cholangitis. In all patients, imaging studies verified the diagnosis of PSC. CC was confirmed histologically in 60 of 62 patients. For patients without available histology the diagnosis of CC was established by clinical, laboratory, radiological and ERC findings. Patients with prior chemotherapy due to CC or obvious metastatic disease were excluded from the study. Demographic data and laboratory values at day of ERC/blood collection were documented.

### Methods

Bile was aspirated by placing a single-use (5 French), standard ERC catheter (without flushing or guidewire cannulation) into the bile duct before contrast material injection. Approximately 0.5 to 5 mL of bile was collected and transferred into a sterile tube. Bile samples were centrifuged (1000 g) and stored at -80 degrees Celsius until miRNA measurement. After centrifugation the serum was stored at -80 degrees for further analysis. The study protocol was approved by the ethics commission of Hannover Medical School and is in accordance with the Declaration of Helsinki. Written informed consent was obtained from all patients.

### MiRNA isolation and quantification

RNA isolation from bile and serum samples was done with miRNeasy Mini Kit (Qiagen, #217004) according to the manufacturer’s protocol. As an internal spiked-in control *Caenorhabditis elegans* (c. elegans) miR-39 was added during the isolation process.

Global profiling of miRNA levels was performed by using a miRNome microRNA Profiler QuantiMir Human PCR Array (#RA660A-1, version 15; BioCat, Heidelberg, Germany) according to manufacturer’s instructions. In brief, poly-A-tailing of miRNAs, annealing of an oligo dT adaptor and then universal reverse transcription was performed. SYBR-Green based Real-time-polymerase chain reaction (PCR) was done with a miRNA-specific and a universal primer. In case of serum, miRNA arrays were performed on pooled RNA samples from six patients for PSC and CC each and a comparison was performed to healthy control patients. For bile, three arrays were performed for the PSC group with pooled RNA from n = 5 patients each and two arrays for the CC group with pools of RNA from n = 4 patients each. Global normalization was done for analysis. Based on strength of regulation and high abundance in the array-analysis distinct miRNAs were chosen for further validation in serum or in bile samples.

For validation RNA was isolated from 83 bile samples (CC n = 19, PSC/CC n = 12, PSC n = 52), 83 serum samples (CC n = 31, PSC n = 40, healthy individuals n = 12) and reverse transcribed with TaqMan MicroRNA Reverse Transcription Kit (Applied Biosystems, #4366597) and specific miRNA Reverse Transcription Primers (Applied Biosystems, #4427975). Real-time-PCR analysis was performed using ABsolute Blue qPCR Mix (Thermo Scientific #AB-4136) and specific miRNA TaqMan Probes (Applied Biosystems, #4427975). Individual miRNA measurements were normalized to spike-in c. elegans controls.

The data discussed in this publication have been deposited in NCBI's Gene Expression Omnibus and are accessible through GEO Series accession number GSE72602 (http://www.ncbi.nlm.nih.gov/geo/query/acc.cgi?acc=GSE72602).

### Statistical analysis

Data were expressed as number/percentages or median with interquartile range (IQR). All data were tested for normality (Shapiro-Wilk test, Kolmogorov-Smirnov test). Non-continuous parameters were analysed by χ^2^-test or fisher’s exact test as appropriate and continuous parameters were analyzed by Mann-Whitney U test. Receiver operating characteristics (ROC) curves were performed to determine the diagnostic accuracy of the different miRNAs. All miRNAs were tested in a logistic regression model to detect an improvement in diagnostic accuracy. In case of missing values, a listwise deletion/omission was done. The cut off points for sensitivity and specificity were calculated with the largest Youden-index. Pearson correlation was used to determine significant correlations between cholestatic parameters and the measured miRNAs. P values < .05 were considered statistically significant. The software used was the SPSS Statistical Package (version 19.0, SPSS Inc, Chicago, Ill)).

## Results

### Analysis of serum miRNAs in patients with PSC, CC and healthy controls

Array-based screening of 1113 miRNAs from RNA pools of six patients with PSC and six patients with CC identified several deregulated miRNAs compared to control patients. The screening patients were matched for gender and age. Additional information on the screening cohort is given in S1 Table. We selected six upregulated miRNAs (miR-126, miR-194, miR-26a, miR-30b, miR-122 and miR-1281) for further analysis based on highest fold change and high detection levels in serum (highlighted in S2 Table).

**Fig 1:**
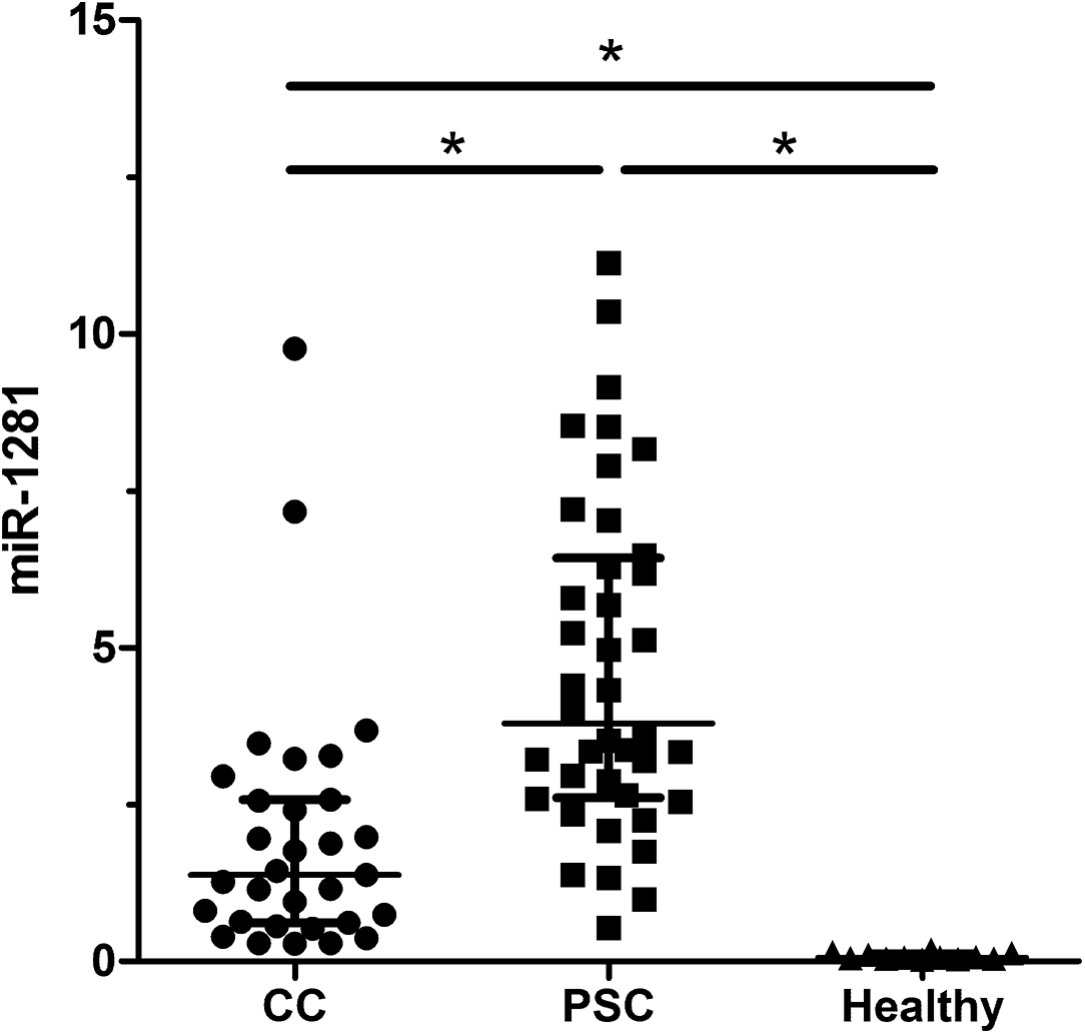
MiRNA analysis in serum. Serum validation analysis for patients with primary sclerosing cholangitis (PSC) (n = 40) and cholangiocarcinoma (CC) (n = 31) revealed differentially expressed miRNAs. Fig 1 shows the result for miR-1281.

**Fig 2:**
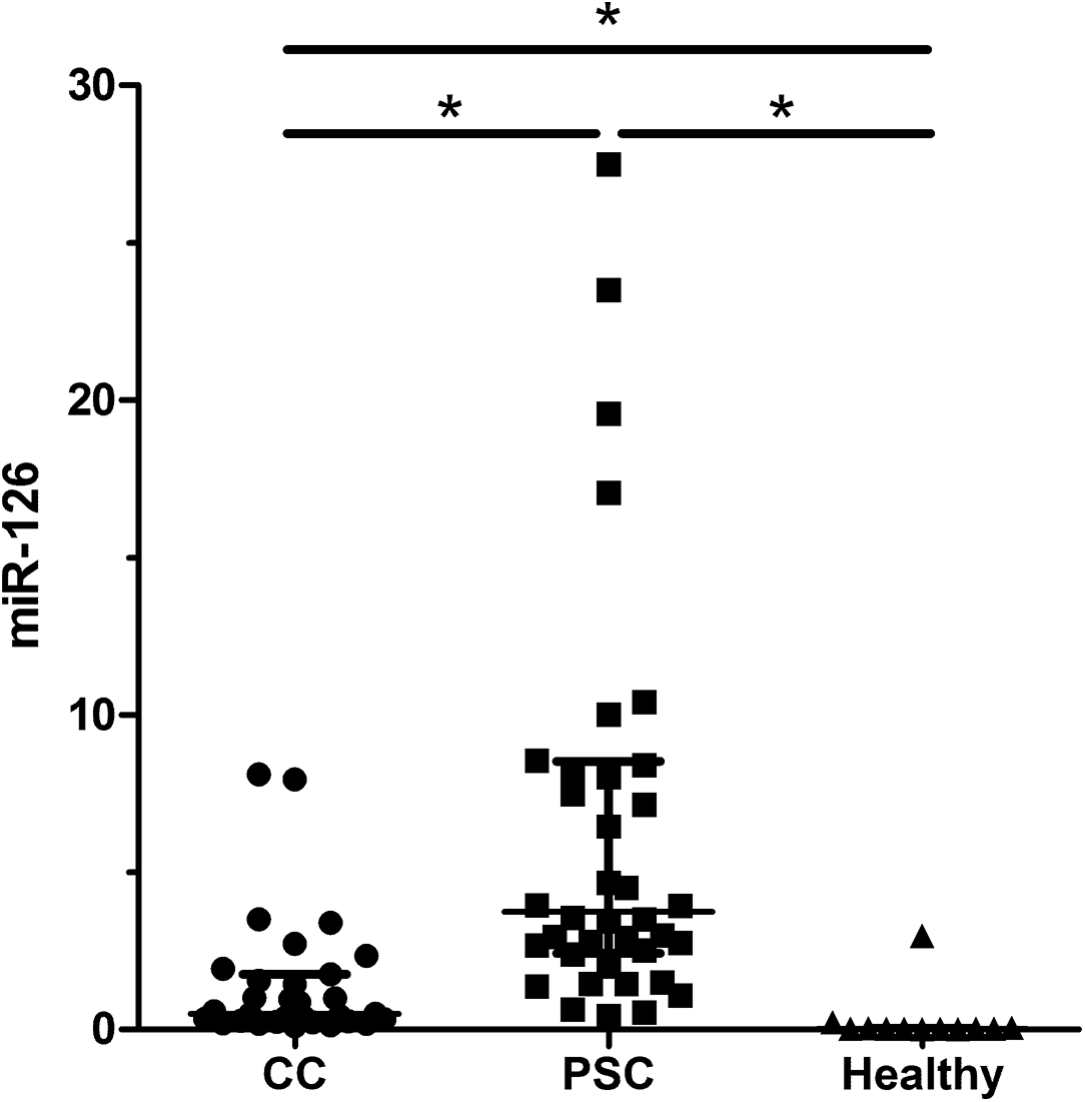
MiRNA analysis in serum. Serum validation analysis for patients with primary sclerosing cholangitis (PSC) (n = 40) and cholangiocarcinoma (CC) (n = 31) revealed differentially expressed miRNAs. Fig 2 shows the result for miR-126.

**Fig 3:**
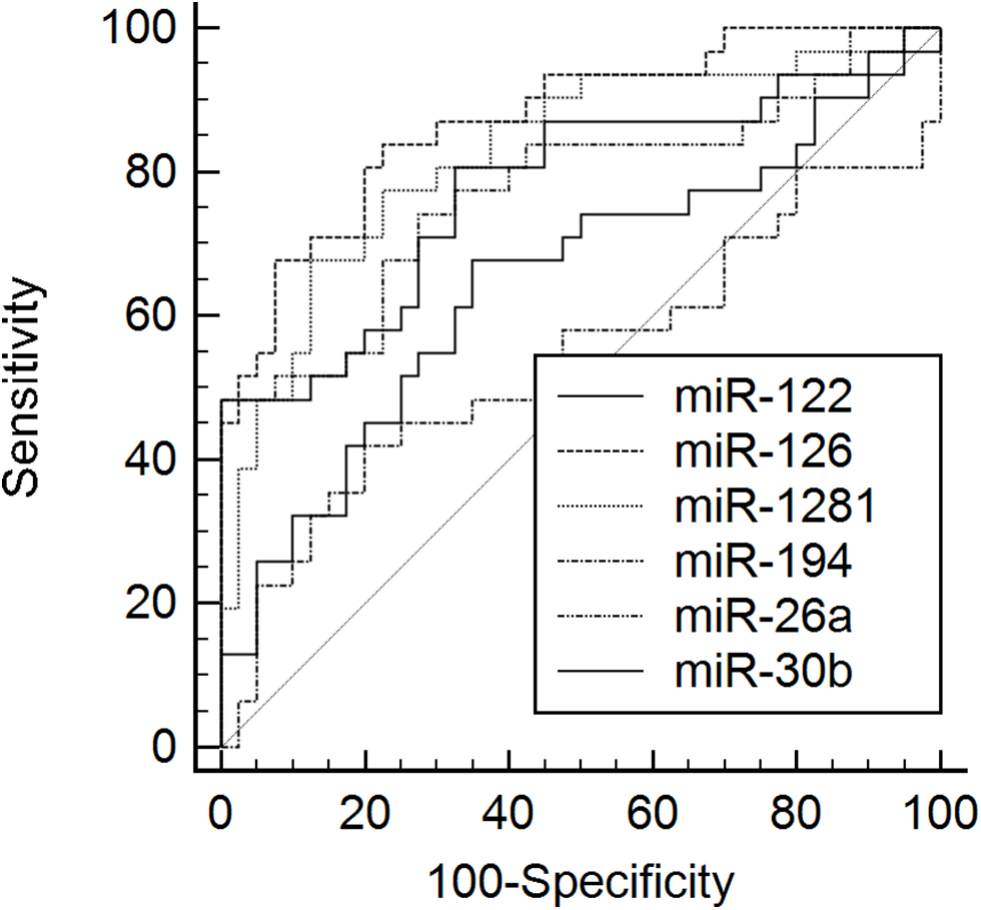
ROC curves for serum miRNAs. Receiver operating characteristics (ROC) curves for the detected serum MIRNAs. MiR-1281 and miR-126 showed the highest area under the curve (AUC) value in ROC curve analysis.

Validation was then performed in 83 serum samples for the aforementioned miRNAs. C. elegans miR-39 values were similar in both groups (PSC and CC) (S1 Fig). Serum analysis revealed significant differences for miR-1281 (p = 0.001), miR-126 (p = 0.001), miR-26a (p = 0.001), miR-30b (p = 0.001) and miR-122 (p = 0.034) between patients with PSC and patients with CC (Figs 1 and 2 (miR-1281, miR-126) and S2–S4 Figs (miR-26a, miR-30b, miR-122)). MiR-1281, miR-126, and miR-26a displayed higher levels in patients with PSC. In addition, miR-30b and miR-122 were also upregulated in patients with PSC, while miR-194 showed no significant difference. All the validated serum miRNAs were significantly higher in the study patients compared to healthy individuals. In order to compare the diagnostic potential of the different miRNAs a ROC curve analysis was performed. The area under the curve (AUC) values for the different miRNAs were as follows; MiR-1281 0.83 (95% confidence interval (CI) 0.72–0.91, p = 0.001), miR-126 0.87 (95% CI 0.77–0.94, p = 0.001), miR-26a 0.78 (95% CI 0.66–0.87, p = 0.001), miR-30b 0.78 (95% CI 0.67–0.87, p = 0.001) and miR-122 0.65 (95% CI 0.53–0.76, p = 0.032), respectively (Fig 3). The sensitivity and specificity values to distinguish between patients with PSC and CC are given in Table 1. A combination of the different miRNAs in a logistic regression model did not significantly improve the diagnostic accuracy (data not shown). Correlation analysis showed no correlation between the miRNAs and cholestatic parameters.

**Table 1:** Serum miRNA analysis. Sensitivity and specificity to distinguish between patients with primary sclerosing cholangitis (PSC) and cholangiocarcinoma (CC) for the differentially expressed miRNAs. The analysis was optimized regarding specificity. Table 1 shows sensitivity, specificity, cut off values, area under the curve (AUC) values with confidence interval (CI) and p-values.

miRNA	cut off	Sensitivity %	Specificity %	AUC	95% CI	p value
miR-1281	≤ 1.44	55	90	0.83	0.72–0.91	0.001
miR-126	≤ 1.0	68	93	0.87	0.77–0.94	0.001
miR-26a	≤ 0.35	52	93	0.78	0.66–0.87	0.001
miR-30b	≤ 0.74	52	88	0.78	0.67–0.87	0.001
miR-122	≤ 0.84	32	90	0.65	0.53–0.76	0.023

Clinical features, demographics and laboratory values of the validation cohorts are presented in Table 2 (Serum) and Table 3 (Bile). Patients with CC displayed higher cholestatic parameters (alkaline phosphatase (AP), gamma-glutamyl transferase (GGT)) compared to patients with PSC (p = 0.001). Carcinoembryonic antigen 19–9 (CA 19–9) was significantly elevated in patients with CC (p = 0.001).

**Table 2:** Clinical features, demographics and laboratory values of the patient cohorts. The aforementioned parameters are presented in Table 2 (Serum). Patients with primary sclerosing cholangitis (PSC) and cholangiocarcinoma (CC) were compared regarding demographics, laboratory and microRNA (miRNA) values. Data were expressed as number or median with interquartile range (IQR). ECC: extrahepatic cholangiocarcinoma; ICC: intrahepatic cholangiocarcinoma; ALT: alanine aminotransferase; AST: aspartate aminotransferase; AP: alkaline phosphatase; GGT: gamma-glutamyl transferase; CRP: C-reactive protein; WBC: white blood cells; CA 19–9: carbohydrate antigen 19–9.

	Cholangiocarcinoma (CC) (n = 31)	Primary sclerosing cholangitis (PSC) (n = 40)	Reference value	p-value
**Gender**	Male 23, Female 8	Male 31, Female 9	−	0.746
**Age** (years)	67 (56–75)	42 (35–51)	−	0.001
**ECC/ICC (n)**	28/3	−	−	−
ALT	60 (30–110)	52 (30–75)	< 45 U/l	0.238
AST	69 (35–137)	47 (38–61)	< 35 U/l	0.107
AP	395 (213–538)	187 (122–304)	40–129 U/l	0.001
GGT	363 (177–694)	169 (66–298)	< 55 U/l	0.001
Bilirubin	31 (10–145)	14 (10–20)	< 2–21 μmol/l	0.045
CRP	23 (6–80)	3 (1–12)	< 8 mg/l	0.001
WBC	8 (6–10)	6 (5–8)	4.4–11.3 /nl	0.142
CA 19–9	308 (68–2483)	27 (8–42)	< 37 kU/l	0.001
**MicroRNAs**				
miR-194	1.97 (0.34–6.61)	2.6 (1.16–5.66)	−	0.562
miR-1281	1.38 (0.61–2.58)	3.79 (2.62–6.38)	−	0.001
miR-126	0.5 (0.26–1.75)	3.75 (2.46–8.48)	−	0.001
miR-122	2.2 (0.42–7.9)	4.59 (2.14–10.91)	−	0.034
miR-26a	0.5 (0.23–1.42)	2.3 (1.08–3.62)	−	0.001
miR-30b	0.74 (0.25–1.69)	2.39 (1.16–4.24)	−	0.001

**Table 3:** Clinical features, demographics and laboratory values of the patient cohorts. The aforementioned parameters are presented in Table 3 (Bile). Patients with primary sclerosing cholangitis (PSC) and cholangiocarcinoma (CC) were compared regarding demographics, laboratory and microRNA (miRNA) values. Data were expressed as number or median with interquartile range (IQR). ECC: extrahepatic cholangiocarcinoma; ICC: intrahepatic cholangiocarcinoma; ALT: alanine aminotransferase; AST: aspartate aminotransferase; AP: alkaline phosphatase; GGT: gamma-glutamyl transferase; CRP: C-reactive protein; WBC: white blood cells; CA 19–9: carbohydrate antigen 19–9.

	Primary sclerosing cholangitis (PSC) (n = 52)	Cholangiocarcinoma Complicating PSC (PSC/CC) (n = 12)	Cholangiocarcinoma (CC) (n = 19)	Reference value	p-value
**Gender**	Male 41, Female 11	Male 9, Female 3	Male 10, Female 9	−	0.198
**Age** (years)	46 (37–52)	49 (37–58)	59 (55–72)	−	0.001
**ECC/ICC (n)**	−	10/2	16/3	−	−
ALT	49 (29–86)	44 (35–176)	43 (28–95)	< 45 U/l	0.714
AST	49 (34–80)	70 (37–214)	50 (36–139)	< 35 U/l	0.428
AP	211 (119–351)	322 (255–1348)	288 (210–483)	40–129 U/l	0.011
GGT	159 (69–310)	502 (103–941)	325 (209–560)	< 55 U/l	0.001
Bilirubin	15 (11–29)	37 (9–310)	15 (8–86)	< 2–21 μmol/l	0.598
CRP	8 (2–19)	9 (4–33)	14 (8–80)	< 8 mg/l	0.067
WBC	6.5 (5.3–8.1)	6.5 (4.8–7.1)	10.2 (5.1–11.9)	4.4–11.3 /nl	0.076
CA 19–9	29 (12–48)	286 (42–567)	104 (47–1869)	< 37 kU/l	0.001
**MicroRNAs**					
miR-132	12.34 (10.14–15.32)	18.71 (10.46–20.48)	5.89 (3.26–11.64)	−	0.001
miR-192	5.57 (4.05–9.14)	10.8 (3.37–15.2)	0 (0–3.93)	−	0.001
miR-194	6.66 (4.28–9.95)	12.15 (5.13–17.1)	0.89 (0–4.73)	−	0.001
miR-215	12.31 (8.9–15.59)	17.26 (8.23–20.79)	4.69 (1.94–10.69)	−	0.001
miR-302b*	18.02 (16.19–20.86)	22.71 (19.98–25.99)	14.03 (10.57–16.18)	−	0.001
miR-412	15.5 (13.73–18.3)	22.02 (18.02–25.96)	12.42 (8.62–15.28)	−	0.001
miR-640	13.94 (12.57–16.65)	20.32 (17.02–23.01)	9.81 (7.37–13.21)	−	0.001
miR-1537	11.38 (9.66–15.91)	18.69 (13.71–21.11)	7.58 (5.77–10.61)	−	0.001
miR-3189	9.84 (8.6–13.83)	15.74 (13.31–19.5)	7.57 (4.06–8.94)	−	0.001

### Analysis of miRNAs from bile of patients with PSC, PSC/CC and CC

PCR-based miRNA arrays were performed from pooled bile RNA samples of patients with PSC and CC to identify deregulated miRNAs. The screening patients were matched for gender and age. Additional information on the screening cohort is given in S3 Table. Five upregulated miRNAs (miR-215, miR-194, miR-132, miR-412, and miR-192) and four downregulated miRNAs (miR-1537, miR-640, miR-302b*, and miR-3189) were chosen for further investigation because of their high deregulation and high abundance in bile (S4 Table). MiR-192 was chosen instead of miR-362-5p due to lower Ct-values in the array analysis.

**Fig 4:**
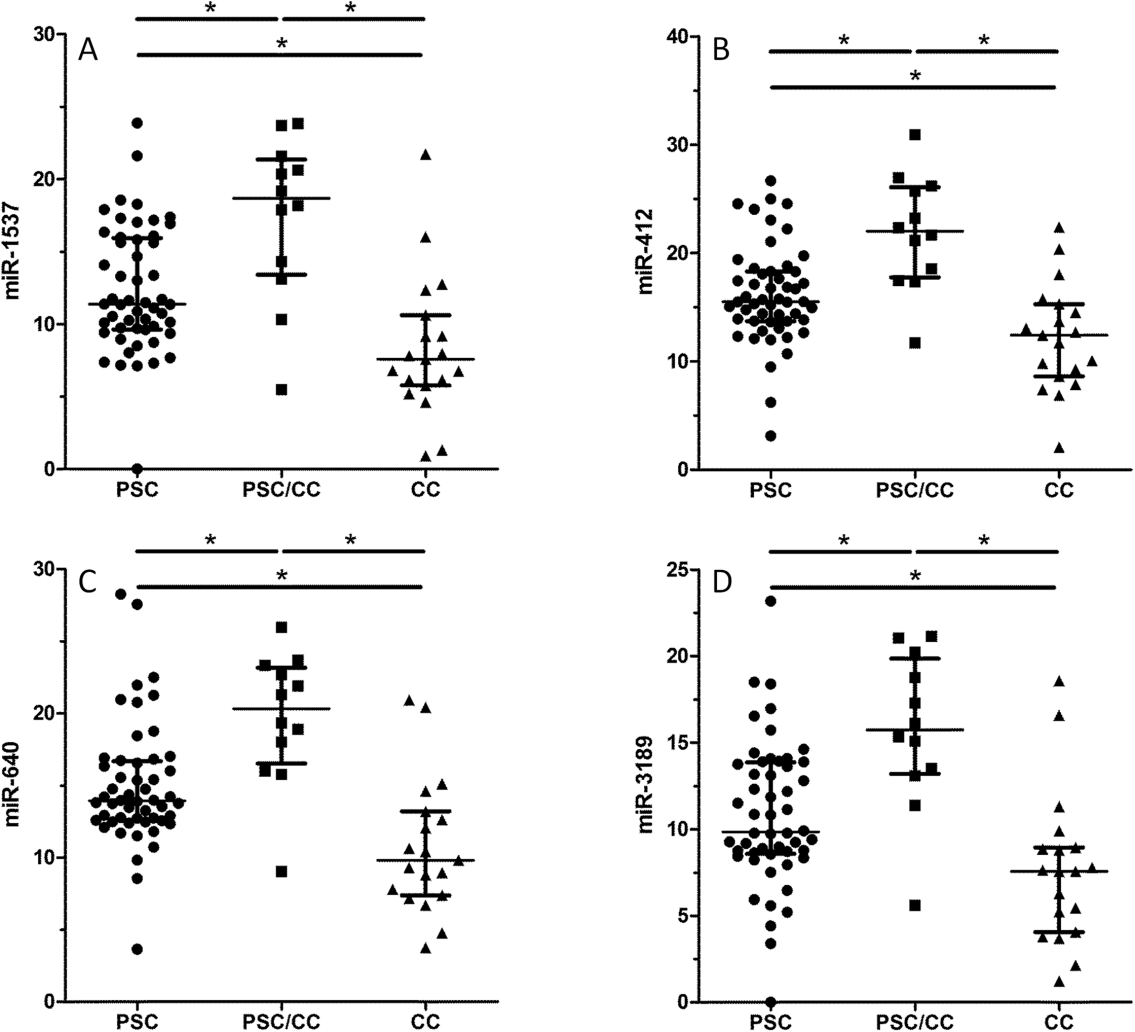
MiRNA analysis in bile. Bile validation analysis for patients with primary sclerosing cholangitis (PSC) (n = 52), cholangiocarcinoma (CC) complicating PSC (PSC/CC) (n = 12) and CC (n = 19) revealed significant differences for the different miRNAs. MiR-1537 (**A**), miR-412 (**B**), miR-640 (**C**) and miR-3189 (**D**) were differentially expressed in patients with PSC and PSC/CC.

C. elegans miR-39 values were equal in both groups (S5 Fig **).** Validation in 83 bile samples revealed significant differences for the investigated miRNAs (Fig 4 and S6 Fig). All miRNAs were significantly higher in patients with PSC or PSC/CC compared to patients with CC (p ≤ 0.05). MiR-412 (p = 0.001), miR-640 (p = 0.001), miR-1537 (p = 0.003) and miR-3189 (p = 0.001) showed a significant difference between patients with PSC and PSC/CC. ROC curve analysis for the comparison between PSC and PSC/CC for the aforementioned miRNAs displayed the following AUC values; MiR-412 0.81 (95% CI 0.69–0.9, p = 0.001), miR-640 0.81 (95% CI 0.69–0.9, p = 0.001), miR-1537 0.78 (95% CI 0.66–0.87, p = 0.003) and miR-3189 0.8 (95% CI 0.68–0.89, p = 0.001). The sensitivity and specificity values to distinguish between patients with PSC and PSC/CC are highlighted in Table 4. The combination of miR-1537 and CA 19–9 in a logistic regression model resulted in an AUC value of 0.91 (95% CI 0.80–0.97, p = 0.001) with a sensitivity of 73% and a specificity of 93%, respectively. Eight out of eleven patients with PSC/CC were correctly classified (one missing CA 19–9 value). Comparison of ROC curves for miR-1537 and CA 19–9 vs. CA 19–9 alone showed a higher AUC value for the combination (0.91 vs. 0.88; p > 0.05). No correlation was found for the miRNAs and cholestatic parameters (S5 Table).

**Table 4:** Bile miRNA analysis. Sensitivity and specificity to distinguish between patients with primary sclerosing cholangitis (PSC) and cholangiocarcinoma complicating PSC (PSC/CC) in bile for the differentially expressed miRNAs. The analysis was optimized regarding specificity. Table 4 shows the sensitivity, specificity, cut off values, area under the curve (AUC) values with confidence interval (CI) and p-values.

MiRNA	cut off	Sensitivity %	Specificity %	AUC	95% CI	p value
miR-412	≥ 22.22	50	89	0.81	0.69–0.9	0.001
miR-640	≥ 21.25	50	92	0.81	0.69–0.9	0.001
miR-1537	≥ 17.38	67	90	0.78	0.66–0.87	0.003
miR-3189	≥ 14.62	67	89	0.8	0.68–0.89	0.001

## Discussion

PSC remains a “black box” in Hepatology. The aetiopathogenesis of PSC is unkown, the clinical course is not predictable and there is no standard therapy [1–3]. Therefore, diagnostic and therapeutic approaches are urgently needed to improve the treatment and prognosis of patients with PSC. Particularly, the early detection of CC in PSC is a clinical challenge and of special interest [6, 8, 13, 14]. Patients with PSC pose a diagnostic dilemma as the risk for hepatobiliary cancer is dramatically increased but only insufficient surveillance tools are available leading to a late diagnosis in the vast majority of patients. Consequently, potential markers for CC diagnosis in PSC are required.

We recently demonstrated that urine and bile proteome analysis differentiates CC from PSC [15, 16]. Another interesting approach is an analysis of miRNA patterns in serum and bile [8, 9]. MiRNAs play a crucial role in gene regulation and are deregulated in pathological conditions. Different miRNAs have been associated with various liver diseases such as hepatocellular carcinoma and cholestatic liver diseases [17–19]. These findings prompted us to further investigate the role of extracellular miRNAs in serum and bile of patients with PSC and/or CC.

To date, there is limited information about the role of miRNAs in biliary diseases and no information of serum miRNAs in PSC. We identified several regulated miRNAs which concentration is higher in PSC and lower in CC (miR-194, miR-1281, miR-126, miR-122, miR-26a and miR-30b). Additionally, these miRNAs are significantly lower in healthy individuals. In the literature, miR-194 is upregulated in patients with pancreatic cancer and is associated with liver and gastric cancer [20–22]. MiR-1281 is downregulated in bladder tumours whereas miR-126 is described to play a critical role in granulocyte colony-stimulating factor-induced hematopoietic progenitor cell mobilization [23, 24]. Moreover, miR-126 inhibits invasion in non-small cell lung carcinoma cell lines and suppresses mesothelioma malignancy [25, 26]. MiR-122 is the foremost liver-related miRNA which is described to play an important role in different liver-associated pathologies [27–29]. Mir-26a inhibits cell proliferation and invasion of cervical cancer cells and is involved in the cell cycle regulation of pituitary adenomas [30, 31]. MiR-30b functions as a tumour suppressor in human colorectal cancer and was identified as one potential candidate miRNA in biliary atresia [32, 33]. These reports show that part of the identified miRNAs have already been described in liver-associated diseases and—if proven in larger studies—may be helpful for the diagnosis of CC in PSC. The diagnosis of CC implies dramatic consequences as CC is only curable by major surgery, e.g. liver transplantation [34]. Hence, a high specificity of a diagnostic test is a prerequisite for clinical application. Several of the detected miRNAs show a considerable overlap between patients with PSC and CC and therefore are mainly of scientific interest. The most promising miRNA in serum analysis was miR-126. The specificity of 93% leads to an incorrect diagnosis in almost one out of ten patients which has to be taken into account and may limit the clinical utility of this miRNA. In addition, the sensitivity of miR-126 shows comparable results to cytology. Further studies are needed to verify the results and to analyze if a combination with other markers or imaging studies may improve the diagnostic yield of miR-126.

*Shigehara et al*. described the potential use of bile miRNAs (esp. miR-9) for the detection of CC [35]. However, miR-9 was not associated with CC in our serum and bile miRNA analysis. This may be an effect of a different normalization. There is a lack of widely accepted standardization methods of miRNA analyses in different body fluids. A general standardization method is urgently needed to provide reliable and reproducible results. Even the selection of the appropriate reference gene for normalization remains unclear [8]. For example, RNA U6 is believed to be a poorer normalizer compared to spiked-in controls of synthetic miRNAs as U6 is less stable and shows a different dynamic of degradation in body fluids [8]. Consequently, we selected c. elegans miR-39 as a normalizer. Moreover, the sample acquisition, processing and storage might differ between centres leading to different sample quality. Our cohort consisted of patients with PSC at risk for CC who generally display chronic inflammation of the bile ducts in contrast to patients with choledocholithiasis. All these circumstances might contribute to the identification of different diagnostic miRNAs in our study compared to *Shigehara et al*.

As the inflammatory and malignant process takes place at the level of the biliary epithelium in patients with PSC and CC we wanted to further analyze the microRNAome in bile. The analysis of miRNAs in different compartments is an interesting approach to provide a mechanistic insight into the pathophysiology of CC and also PSC. In our study, the analyzed miRNAs were significantly elevated in bile of patients with PSC compared to CC. We identified four miRNAs (miR-640, miR-3189, miR-1537 and miR-412) which differed significantly between patients with PSC and PSC/CC. In the literature, limited data is available for the detected miRNAs. MiR-640 was differentially expressed in a distinct cytogenetic subgroup of chronic lymphocytic leukemia and downregulated in tumour samples of serous ovarian carcinoma [36, 37]. MiR-412 was upregulated in squamous cell lung carcinoma tissues compared with normal lung tissues [38]. Besides, miR-412 was utilized in a diagnostic panel of several miRNAs to distinguish hypoxic and toxic liver injury [39]. Interestingly, the aforementioned miRNAs showed higher levels in PSC/CC vs. PSC but lower levels in CC vs. PSC/CC. This counterintuitive finding might be due to the divergent aetiopathogenesis of CC in PSC compared to CC without underlying chronic biliary disease. However, this assumption remains speculative and cannot be conclusively answered by this study. This also accounts for the detection of different miRNAs in serum and in bile. We hypothesize that the detected miRNAs in serum might be an epiphenomenon of CC development. In addition, validation analysis is expensive and only a limited number of target miRNAs can be tested. Basis for the selection of the miRNAs is the array analysis which might be influenced by selection bias and can lead to contradictory results between array and validation analysis. As ERC is an invasive procedure with potential life-threatening complications no bile specimens from healthy individuals were available which limits the results from bile analysis.

*Li et al*. identified that miRNA-laden extracellular vesicles are present in human bile and can be used for CC diagnosis [8]. In their comprehensive study the authors concluded that miRNAs in human bile derive from free-floating cells as well as biliary extracellular vesicles. In contrast to the miRNAs derived from vesicles the miRNA species from cells were instable and thus not suitable for diagnostic approaches [8].

In addition, *Li et al*. delineated a different miRNA pattern to detect CC in bile [8, 35]. This result is plausible as the miRNA species were solely isolated from extracellular vesicles without consideration of the soluble fraction of bile. Recent publications suggest that a significant additional amount of extracellular miRNAs are not present in vesicles [40, 41]. Moreover, *Li et al*. only included a small number of patients with PSC in their control group which may also contribute to the difference in the miRNA pattern. In our analysis, miR-1537 and miR-3189 in bile showed encouraging results for patients with PSC/CC which legitimatizes future studies.

As cholestasis may lead to an alteration of miRNA measurement, we performed correlation analysis to exclude a significant influence of elevated cholestatic parameters. No relevant correlation was found for the miRNAs indicating that cholestasis in CC does not interfere with the miRNA measurement.

Unfortunately, we did not have paired blood and bile samples from each patient. Consequently, a combination of blood and bile markers to establish a combined marker panel is not feasible. Moreover, it is difficult to acquire abundant samples from patients with PSC/CC. However, twelve bile samples from PSC/CC are a valuable cohort for the detection of differentially expressed miRNAs. Nevertheless, future studies are warranted to prospectively validate our findings and to evaluate if a combination of different markers (e.g. miRNAs and CA 19–9) may improve the diagnosis of CC in PSC.

In summary, our study delineates the miRNA profile in serum and bile of patients with PSC and CC and adds new knowledge to the developing field of miRNA analysis. The identified dysregulated miRNAs might be used for diagnostic purposes but have to be validated in larger cohorts. Pathophysiological aspects of these miRNAs have to be addressed in future studies.

## Supporting Information

S1 Fig C. elegans miR-39 in PSC and CC (serum). Comparison of c.elegans miR-39 values in patients with cholangiocarcinoma (CC) and control patients (PSC) in serum. No significant difference was detected between both cohorts (ns).
(TIF)

S2 Fig MiRNA analysis in serum. Serum validation analysis for patients with primary sclerosing cholangitis (PSC) (n = 40) and cholangiocarcinoma (CC) (n = 31) revealed differentially expressed miRNAs. S2 Fig shows the result for miR-30b.
(TIF)

S3 Fig MiRNA analysis in serum. Serum validation analysis for patients with primary sclerosing cholangitis (PSC) (n = 40) and cholangiocarcinoma (CC) (n = 31) revealed differentially expressed miRNAs. S3 Fig shows the result for miR-26a.
(TIF)

S4 Fig MiRNA analysis in serum. Serum validation analysis for patients with primary sclerosing cholangitis (PSC) (n = 40) and cholangiocarcinoma (CC) (n = 31) revealed differentially expressed miRNAs. S4 Fig shows the result for miR-122.
(TIF)

S5 Fig C. elegans miR-39 in PSC and CC (bile) Comparison of c.elegans miR-39 values in patients with cholangiocarcinoma (CC) and control patients (PSC) in bile. No significant difference was detected between both cohorts (ns).
(TIF)

S6 Fig MiRNA analysis in bile. Bile validation analysis for patients with primary sclerosing cholangitis (PSC) (n = 52), cholangiocarcinoma (CC) complicating PSC (PSC/CC) (n = 12) and CC (n = 19) revealed significant differences for the different miRNAs. The results are shown for miR-192 (**A**), miR-215 (**B**), miR-302b* (**C**), miR-132 (**D**) and miR-194 (**E**).
(TIF)

S1 Table Demographics and laboratory parameters of the screening cohort (serum analysis). RNA pools of six patients with primary sclerosing cholangitis (PSC) and six patients with cholangiocarcinoma (CC) identified several deregulated miRNAs compared to healthy control patients. The screening patients were matched for gender and age. Demographics and laboratory values of the screening cohort are presented in **S1 Table**. Data were expressed as number or median with interquartile range (IQR). ALT: alanine aminotransferase; AST: aspartate aminotransferase; AP: alkaline phosphatase; GGT: gamma-glutamyl transferase; CRP: C-reactive protein; WBC: white blood cells; CA 19–9: carbohydrate antigen 19–9.
(DOCX)

S2 Table Array analysis for serum miRNAs. Differentially expressed microRNAs with fold changes in serum of patients with primary sclerosing cholangitis (PSC) (A) and CC (B) compared to healthy control patients. The miRNAs miR-126, miR-26a, miR-1281, miR-30b, miR-194, miR-122 and miR-26a were chosen for further validation.
(DOCX)

S3 Table Demographics and laboratory parameters of the screening cohort (bile analysis). RNA pools of 15 patients with primary sclerosing cholangitis (PSC) and eight patients with cholangiocarcinoma (CC) identified several deregulated miRNAs in bile. The screening patients were matched for gender and age. Demographics and laboratory values of the screening cohort are presented in **S3 Table**. Data were expressed as number or median with interquartile range (IQR). ALT: alanine aminotransferase; AST: aspartate aminotransferase; AP: alkaline phosphatase; GGT: gamma-glutamyl transferase; CRP: C-reactive protein; WBC: white blood cells; CA 19–9: carbohydrate antigen 19–9.
(DOCX)

S4 Table Array analysis for bile miRNAs (PSC and CC). Differentially expressed microRNAs with fold changes in bile of patients with primary sclerosing cholangitis (PSC) and cholangiocarcinoma (CC). The upregulated miRNAs miR-215, miR-194, miR-132, miR-412, miR-192 and downregulated miRNAs miR-1537, miR-640, miR-302b* and miR-3189 were chosen for further validation. MiR-192 was chosen instead of miR-362-5p due to lower Ct-values in the array analysis.
(DOCX)

S5 Table Correlation of bile miRNAs and bilirubin. All validated miRNAs in bile were analyzed regarding correlation to bilirubin. No relevant correlation was detected. R: correlation coefficient.
(DOC)
