# Supplementary figures and images for: MicroRNAs in Serum and Bile of Patients with Primary Sclerosing Cholangitis and/or Cholangiocarcinoma
Source: PLoS One. 2015 Oct 2;10(10):e0139305. doi: 10.1371/journal.pone.0139305 (PMC4591993; doi:10.1371/journal.pone.0139305)

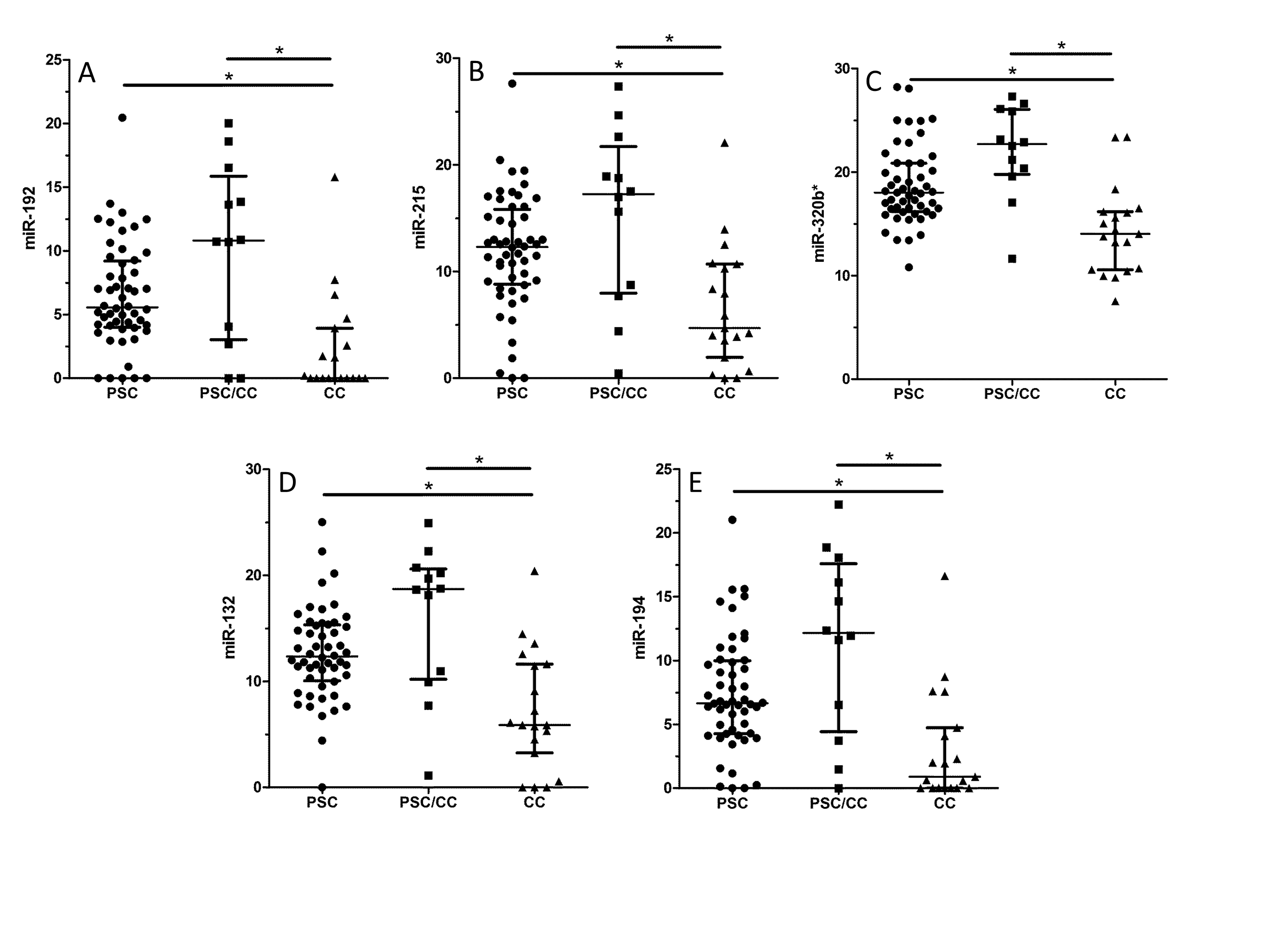

Supplement: S6 Fig — Bile validation analysis for patients with primary sclerosing cholangitis (PSC) (n = 52), cholangiocarcinoma (CC) complicating PSC (PSC/CC) (n = 12) and CC (n = 19) revealed significant differences for the different miRNAs. The results are shown for miR-192 (A), miR-215 (B), miR-302b* (C), miR-132 (D) and miR-194 (E). (TIF) [file pone.0139305.s006.tif]
