# Supplementary material for: MicroRNAs in Serum and Bile of Patients with Primary Sclerosing Cholangitis and/or Cholangiocarcinoma
Source: PLoS One. 2015 Oct 2;10(10):e0139305. doi: 10.1371/journal.pone.0139305 (PMC4591993; doi:10.1371/journal.pone.0139305)
Supplement: S1 Table — RNA pools of six patients with primary sclerosing cholangitis (PSC) and six patients with cholangiocarcinoma (CC) identified several deregulated miRNAs compared to healthy control patients. The screening patients were matched for gender and age. Demographics and laboratory values of the screening cohort are presented in S1 Table. Data were expressed as number or median with interquartile range (IQR). ALT: alanine aminotransferase; AST: aspartate aminotransferase; AP: alkaline phosphatase; GGT: gamma-glutamyl transferase; CRP: C-reactive protein; WBC: white blood cells; CA 19–9: carbohydrate antigen 19–9. (DOCX) [file pone.0139305.s007.docx]

|  | **Cholangiocarcinoma (CC) (n = 6)** | **Primary sclerosing cholangitis (PSC) (n = 6)** | **Reference value** | **p-value** |
| --- | --- | --- | --- | --- |
| Gender | M4, F2 | M4, F2 | − | 0.248 |
| Age | 53 (45-55) | 54 (44-65) | − | 0.818 |
| **Laboratory values** |  |  |  |  |
| ALT | 106 (60-110) | 24 (22-50) | < 45 U/l | 0.151 |
| AST | 63 (49-90) | 34 (29-46) | < 35 U/l | 0.082 |
| AP | 432 (186-702) | 120 (87-477) | 40-129 U/l | 0.132 |
| GGT | 496 (186-1302) | 72 (36-314) | < 55 U/l | 0.041 |
| Bilirubin | 77 (7-175) | 12 (8-19) | < 2-21 µmol/l | 0.589 |
| CRP | 35 (6-77) | 2 (2-5) | < 8 mg/l | 0.082 |
| WBC | 6.7 (5.4-9.3) | 6.2 (6-7.8) | 4.4 - 11.3 /nl | 1.000 |
| CA 19-9 | 374 (354-22449) | 34 (8-113) | < 37 kU/l | 0.056 |

**Supplementary Table 1**
