# Supplementary material for: MicroRNAs in Serum and Bile of Patients with Primary Sclerosing Cholangitis and/or Cholangiocarcinoma
Source: PLoS One. 2015 Oct 2;10(10):e0139305. doi: 10.1371/journal.pone.0139305 (PMC4591993; doi:10.1371/journal.pone.0139305)
Supplement: S2 Table — Differentially expressed microRNAs with fold changes in serum of patients with primary sclerosing cholangitis (PSC) (A) and CC (B) compared to healthy control patients. The miRNAs miR-126, miR-26a, miR-1281, miR-30b, miR-194, miR-122 and miR-26a were chosen for further validation. (DOCX) [file pone.0139305.s008.docx]

**S2 Table (A)**

| **Serum miRNA** | **Fold change between PSC and healthy controls** |
| --- | --- |
| **miR-126** | **21.75** |
| **miR-26a** | **10.40** |
| **miR-1281** | **3.53** |
| **miR-30b** | **3.40** |
| miR-221 | 2.26 |
| miR-494 | 2.49 |
| miR-125a-5p | 0.07 |
| miR-101 | 0.12 |
| miR-1275 | 0.15 |
| miR-1183 | 0.17 |
| miR-181a | 0.18 |
| miR-2861 | 0.25 |
| miR-451 | 0.28 |
| miR-1915 | 0.3 |
| miR-1268 | 0.31 |
| miR-320c | 0.34 |
| miR-636 | 0.36 |
| miR-320b | 0.37 |
| miR-1225-3p | 0.37 |
| miR-181c | 0.39 |
| miR-320a | 0.41 |
| miR-19b | 0.43 |
| miR-1308 | 0.44 |
| miR-574-5p | 0.45 |
| miR-135b | 0.45 |
| miR-20a | 0.49 |

**S2 Table (B)**

| **Serum miRNA** | **Fold change between CC and healthy controls** | |
| --- | --- | --- |
| **miR-194** | | **12.58** |
| **miR-30b** | | **7.01** |
| **miR-122** | | **4.69** |
| **miR-26a** | | **3.74** |
| miR-1979 | | 3.37 |
| miR-30a | | 3.02 |
| miR-142-3p | | 2.68 |
| miR-21 | | 2.24 |
| miR-324-5p | | 2.18 |
| miR-1275 | | 0.15 |
| miR-636 | | 0.19 |
| miR-125a-5p | | 0.21 |
| miR-181a | | 0.24 |
| miR-92b | | 0.24 |
| miR-2861 | | 0.24 |
| miR-1225-3p | | 0.25 |
| miR-1268 | | 0.25 |
| miR-1247 | | 0.28 |
| miR-23b | | 0.29 |
| miR-1915 | | 0.33 |
| miR-671-3p | | 0.34 |
| miR-19b | | 0.35 |
| miR-181c | | 0.36 |
| miR-1978 | | 0.40 |
| miR-1974 | | 0.40 |
| miR-320b | | 0.41 |
| miR-320c | | 0.42 |
| miR-1183 | | 0.42 |
| miR-191 | | 0.44 |
| miR-1228 | | 0.46 |
| miR-486-5p | | 0.48 |
