# Supplementary material for: MicroRNAs in Serum and Bile of Patients with Primary Sclerosing Cholangitis and/or Cholangiocarcinoma
Source: PLoS One. 2015 Oct 2;10(10):e0139305. doi: 10.1371/journal.pone.0139305 (PMC4591993; doi:10.1371/journal.pone.0139305)
Supplement: S3 Table — RNA pools of 15 patients with primary sclerosing cholangitis (PSC) and eight patients with cholangiocarcinoma (CC) identified several deregulated miRNAs in bile. The screening patients were matched for gender and age. Demographics and laboratory values of the screening cohort are presented in S3 Table. Data were expressed as number or median with interquartile range (IQR). ALT: alanine aminotransferase; AST: aspartate aminotransferase; AP: alkaline phosphatase; GGT: gamma-glutamyl transferase; CRP: C-reactive protein; WBC: white blood cells; CA 19–9: carbohydrate antigen 19–9. (DOCX) [file pone.0139305.s009.docx]

|  | **Cholangiocarcinoma (CC) (n = 8)** | **Primary sclerosing cholangitis (PSC) (n = 15)** | **Reference value** | **p-value** |
| --- | --- | --- | --- | --- |
| Gender | M4, F4 | M9, F6 | **−** | 0.532 |
| Age | 58 (52-65) | 51 (48-64) | **−** | 0.357 |
| **Laboratory values** |  |  |  |  |
| ALT | 75 (56-105) | 50 (35-70) | < 45 U/l | 0.034 |
| AST | 48 (35-73) | 45 (34-67) | < 35 U/l | 0.875 |
| AP | 428 (352-528) | 125 (80-222) | 40-129 U/l | 0.001 |
| GGT | 285 (182-373) | 123 (76-200) | < 55 U/l | 0.016 |
| Bilirubin | 61 (29-74) | 23 (17-33) | < 2-21 µmol/l | 0.008 |
| CRP | 20 (12-38) | 5 (3-22) | < 8 mg/l | 0.115 |
| WBC | 6.9 (6.3-9.8) | 5.6 (4.9-7.7) | 4.4 - 11.3 /nl | 0.149 |
| CA 19-9 | 32 (12-94) | 9 (5-23) | < 37 kU/l | 0.056 |

**Supplementary Table 3**
