# Supplementary material for: MicroRNAs in Serum and Bile of Patients with Primary Sclerosing Cholangitis and/or Cholangiocarcinoma
Source: PLoS One. 2015 Oct 2;10(10):e0139305. doi: 10.1371/journal.pone.0139305 (PMC4591993; doi:10.1371/journal.pone.0139305)
Supplement: S4 Table — Differentially expressed microRNAs with fold changes in bile of patients with primary sclerosing cholangitis (PSC) and cholangiocarcinoma (CC). The upregulated miRNAs miR-215, miR-194, miR-132, miR-412, miR-192 and downregulated miRNAs miR-1537, miR-640, miR-302b* and miR-3189 were chosen for further validation. MiR-192 was chosen instead of miR-362-5p due to lower Ct-values in the array analysis. (DOCX) [file pone.0139305.s010.docx]

| **microRNAs in bile** | **Fold changes in bile of PSC and CC** |
| --- | --- |
| **miR-215** | 11.39 |
| **miR-194** | 5.85 |
| **miR-132** | 5.56 |
| **miR-412** | 5.24 |
| miR-362-5p | 4.94 |
| **miR-192** | 4.88 |
| miR-489 | 4.66 |
| miR-3138 | 4.35 |
| miR-30b | 4.34 |
| **miR-1537** | 0.02 |
| **miR-640** | 0.04 |
| **miR-302b*** | 0.05 |
| **miR-3189** | 0.06 |
| miR-877 | 0.06 |
| miR-665 | 0.07 |
| miR-671-3p | 0.07 |
| miR-769-3p | 0.07 |
| miR-2114 | 0.08 |
| miR-1287 | 0.09 |
| miR-766 | 0.11 |
| miR-218-1* | 0.12 |
| miR-671-5p | 0.12 |
| miR-885-3p | 0.13 |
| miR-3185 | 0.14 |
| miR-770-5p | 0.14 |
| miR-664 | 0.14 |
| miR-3190-3p | 0.15 |
| miR-193b | 0.15 |
| miR-200a* | 0.16 |
| miR-3190-5p | 0.17 |
| miR-661 | 0.17 |
| miR-621 | 0.17 |
| miR-3131 | 0.17 |
| miR-675 | 0.18 |
| miR-3164 | 0.18 |
| miR-769-5p | 0.18 |
| miR-4322 | 0.20 |
| miR-708 | 0.21 |
| miR-3182 | 0.22 |
| miR-4290 | 0.22 |
| miR-875-3p | 0.23 |
