# Supplementary material for: MicroRNAs in Serum and Bile of Patients with Primary Sclerosing Cholangitis and/or Cholangiocarcinoma
Source: PLoS One. 2015 Oct 2;10(10):e0139305. doi: 10.1371/journal.pone.0139305 (PMC4591993; doi:10.1371/journal.pone.0139305)
Supplement: S5 Table — All validated miRNAs in bile were analyzed regarding correlation to bilirubin. No relevant correlation was detected. R: correlation coefficient. (DOC) [file pone.0139305.s011.doc]

|  | **Bilirubin** | **p-value** |
| --- | --- | --- |
| **miR-132** | r = 0.06 | 0.63 |
| **miR-192** | r = 0.05 | 0.7 |
| **miR-194** | r = 0.04 | 0.74 |
| **miR-215** | r = 0.02 | 0.87 |
| **miR-302b*** | r = 0.08 | 0.56 |
| **miR-412** | r = 0.11 | 0.39 |
| **miR-640** | r = 0.14 | 0.3 |
| **miR-1537** | r = 0.14 | 0.3 |
| **miR-3189** | r = 0.15 | 0.25 |
